# Voltammetric Determination of Cysteine (2-amino-3-mercaptopropanoic acid, CySH) by Means of 4,4′-biphenol as a Homogeneous Mediator

**DOI:** 10.22037/ijpr.2019.1100829

**Published:** 2019

**Authors:** Mehrnoush Kamali, Zeinab Pourghobadi

**Affiliations:** *Department of Chemistry, Khorramabad Branch, Islamic Azad University, Khorramabad, Iran.*

**Keywords:** Cysteine, 4, 4′-biphenol, Homogeneous mediator, Voltammetric determination

## Abstract

In the present research, 4,4′-biphenol was used as a homogeneous mediator for determining cysteine (CySH) on a Glassy Carbon Electrode (GCE). To describe the electrochemical properties of 4,4′-biphenol and to examine its electrocatalytic impacts on cysteine oxidation, both Cyclic Voltammetry (CV) and Linear Sweep Voltammetry (LSV) were employed. Our findings revealed that 4,4′-biphenol could significantly accelerate the reactions related to electron transfer to CySH. Moreover, the diffusion coefficient of CySH and its reaction with the catalytic constant of 4,4′-diphenoquinone was estimated via chronoamperometry technique. The results showed that cysteine concentration range of 10-1000 μM led to linear increases in the oxidation peaks, thus to providing a detection of 0.99 μM with R² = 0.993. A Relative Standard Deviation (RSD) of 2.5% was achieved after performing 7 cysteine replicates (100 μM), and CySH was successfully determined in real serum samples through the proposed approach.

## Introduction

Cysteine (2-amino-3-mercaptopropanoic acid, CySH) is an important amino acid widely used in the medicine and food additives. It plays a leading role in the production of collagen in the skin texture to provide its elasticity (1). CySH is also of pivotal importance in protein synthesis, detoxification, autooxidative properties, and diverse metabolic functions (2). Morover, it is important for the metabolism of essential biochemicals, including coenzymes, heparin, and biotin (3). Therefore, the precise measurement of CySH can be significant in pharmaceutical industry. Spectrophotometry (4-5), spectrofluorimetry (6), High-Performance Liquid Chromatography (HPLC) (7-8), chemiluminescence (9), and Gas Chromatography-Mass Spectrometry (GC–MS) (10-11) provide some different approaches to the determination of plasma CySH concentration, which has been recently developed for pharmaceuticals. However, these methods are mostly time-consuming for sample preparation and analysis and expensive in terms of solvent usage and maintenance of devices. Alternatively, electrochemical techniques have attracted more attention for determining pharmaceutical compounds as they are simple, rapid, and precise and can be used for a wide range of detections at relatively low costs. However, CySH is difficulty detected since conventional electrodes are highly sensitive, while being involved in very slow electron-transfer kinetics at high overpotentials despite the fact that it is an electroactive molecule. A possible solution to thsese challenges is that to modify electrodes with the high level of selectivity and analyses (12-21). In this regard, a broad spectrum of mediators were reported to analyze environmental, pharmaceutical, and electro-active materials, several of which are nanoparticles (22), carbon nanotubes (23), nanocomposites (24), and enzyme-based biosensors (25). As reported in the literature (26-28), many analytical approaches to the electrocatalytic oxidation of 4,4′-biphenol have been developed in recent years for determining such thiols as captopril, N-acetylcysteine, and glutathione. The present study dealt with the development and validation of a new sensitive electrocatalytic approach by using 4,4′-biphenol as a homogeneous mediator for promoting CySH oxidation and detection in the plasma samples and pharmaceuticals. To this goal, Cyclic Voltammetry (CV), Linear Sweep Voltammetry (LSV), and chronoamperometry were employed to provide a description of the electrochemical properties of 4,4′-biphenol and examination of its electrocatalytic impacts on CySH oxidation besides calculation of such kinetic parameters as the catalyst reaction constant.

## Experimental

*Apparatus and Chemicals*

By means of NOVA1.11 software, a potentiostat/galvanostat Autolab model PGSTAT 204 (Eco Chemie, Utrecht, Netherlands) was used for electrochemical experiments. The reference electrode consisted of an Ag/AgCl electrode (KCl_3_M) for using the 3- electrode cell. A GCE and a Pt wire were used as the working electrode and the counter electrode, respectively. The glassy carbon electrode was obtained from the AZAR electrode, and the Ag/AgCl electrode and Pt wire were obtained from Metrohm. For pH measurements, moreover, the Metrohm model 827 pH/mV meter was used.

Cysteine and 4,4′-biphenol were obtained from Sigma-Aldrich. Other chemicals were of analytical reagent grade purchased from Merck Company. By dissolving an optimally precise amount of CySH, a 1.0 × 10^−2^ mol L ^−1^ stock solution was freshly prepared. Other fresh solutions were prepared by diluting the optimal stock solution with distilled water. Every experiment was conducted through the application 0.1 M phosphate buffer solutions with various pH values at 25 ± 1 °C.

*Analytical Procedure*

The glassy carbon electrode CySH was measured according to the method reported in a previous work (26) by polishing with an alumina fine powder (0.05 μm) in water slurry and with a polishing cloth. Afterwards, it was ultrasonically washed in ethanol and water, respectively. The electrodes were dipped into a solution of 4,4′-biphenol and buffer in one of the experiments. The potential was swept from -0.2 to + 0.5 V versus Ag/AgCl at the scanning rate of 10 mVs^−1^. While CySH was present as the sample solution, the above procedure was repeated.

*Real sample Preparation*

CySH (1 mL, 0.01 mol L^-1^) spiked in the blood serum sample, and then was centrifuged. The supernatant was diluted 50 times with water. Finally, the standard addition method was used. Two milliliter of the solution and 8.0 mL of the buffer (pH 10.0) were used for the analysis. Obtained from relevant calibration equations, the calibration curve method was used in order to get the quantitations.

**Scheme 1:**
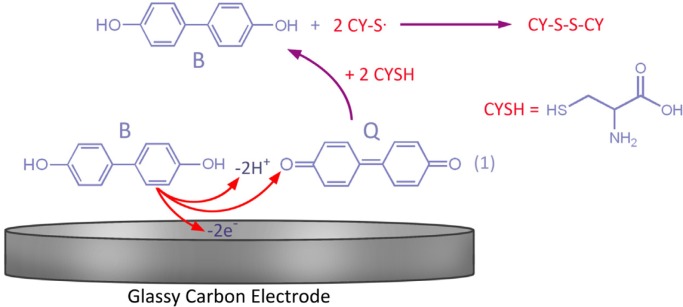
Proposed mechanism for the electrochemical oxidation of 4,4′-biphenol in the presence of CySH

**Figure 1:**
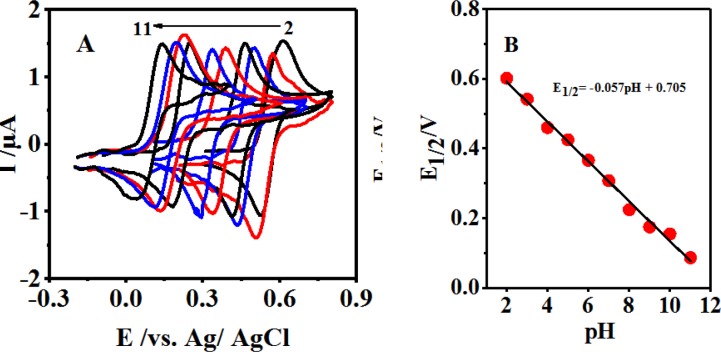
(A) Cyclic voltammograms of 0.1 mM 4,4′-biphenol in buffer solution. With different pH values in the range of 2-11 at a glassy carbon electrode. Scan rate 10 mV/sec. t = 25 ± 1 ºC. (B) Variation of E1/2 *vs*. various pH

**Figure 2:**
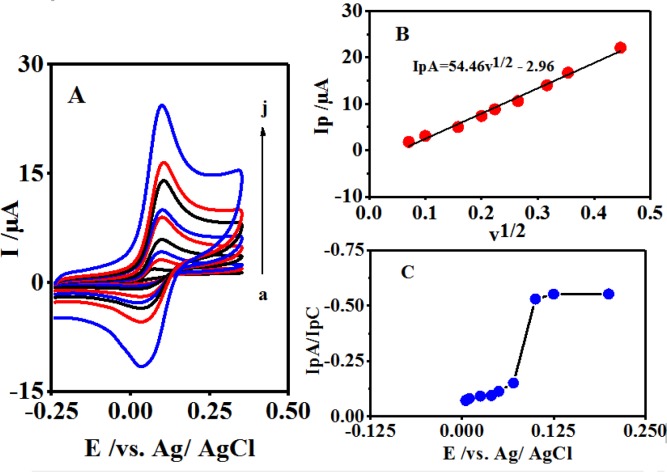
(A) Typical cyclic voltammograms of 0.1 mM 4,4′-biphenol in the presence of CySH (0.5 mM), in buffer solution (pH 10.0). Scan rates are: 5, 10, 25,40,50,70,100,125,150 and 200 mV/sec, respectively. (B) The plot of IpA *vs.* v^1/2^ in the range of 5-200 mV/sec^-1^. (C) (I_pC1_/I_pA1_).

**Figure 3:**
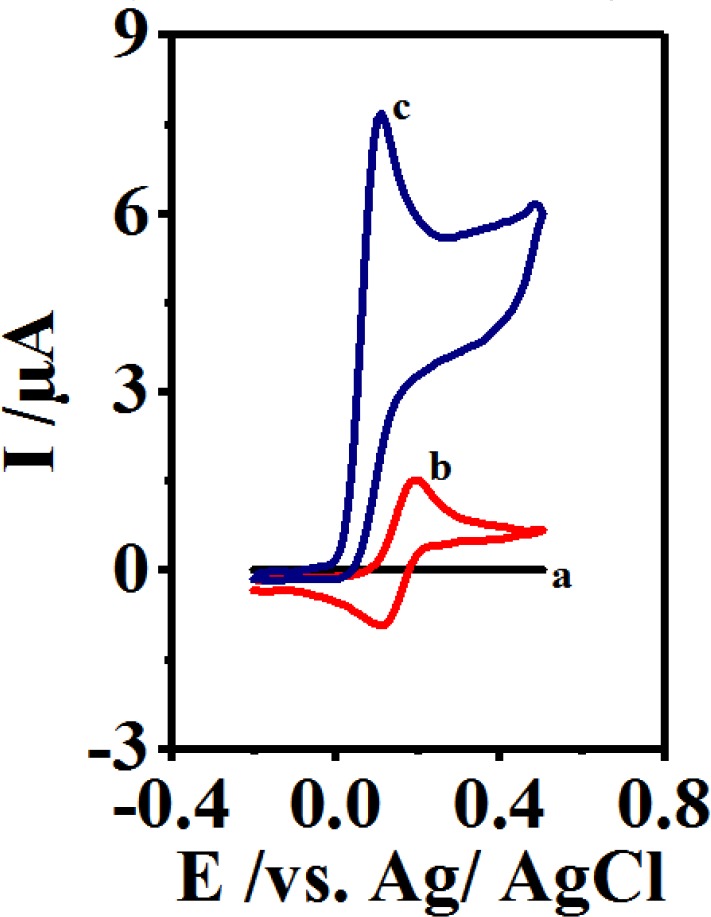
(a) Cyclic voltammogram of 0.1 mM 4,4′-biphenol in the absence CySH. (b) Cyclic voltammogram of 4,4′-biphenol in the presence CySH (2 mM) and (c) cyclic voltammogram 2 mM CySH in the absence of 4,4′-biphenol in buffer solution, pH 10 at a scan rate of 10 mV/sec. t = 25 ± 1 ºC

**Figure 4:**
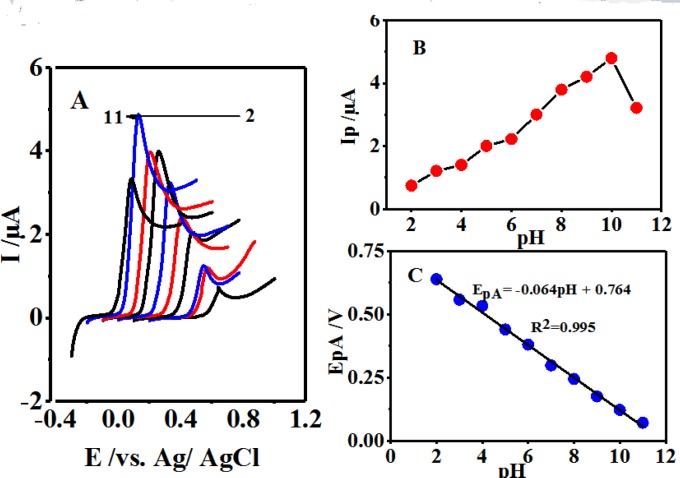
(A) Linear sweep voltammograms of 0.1 mM 4,4′-biphenol in the presence of CySH (0.5 mM) in buffer solutions with different pH values at a glassy carbon electrode. Scan rate 10 mV/sec. (B) Effect of pH on the oxidation peak current (IpA). (C) Effect of pH on the oxidation peak current (EpA). t = 25 ± 1 ºC

**Figure 5:**
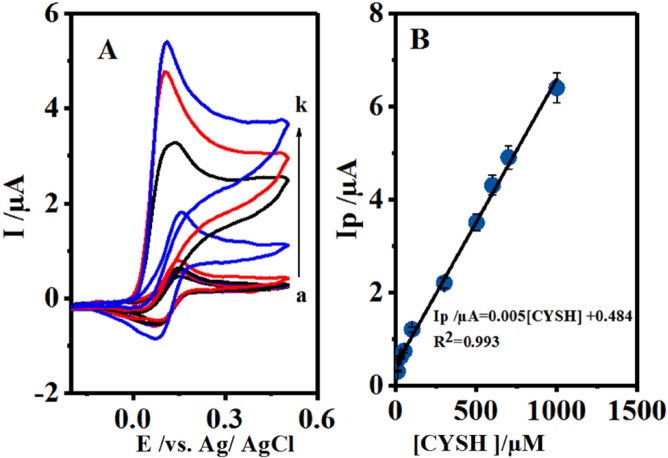
(A) Cyclic voltammograms 0.1 mM 4,4′-biphenol in the presence of different concentration of CySH. Concentration of CySH from a to j are: 10.0, 20.0, 30.0, 50.0, 100.0, 300.0, 500.0, 600.0, 700 and 1000.0 μM. Scan rate: 10 mV/sec. (B) Plot of the anodic peak current versus concentration of CySH. t = 25 ± 1 ºC

**Figure 6:**
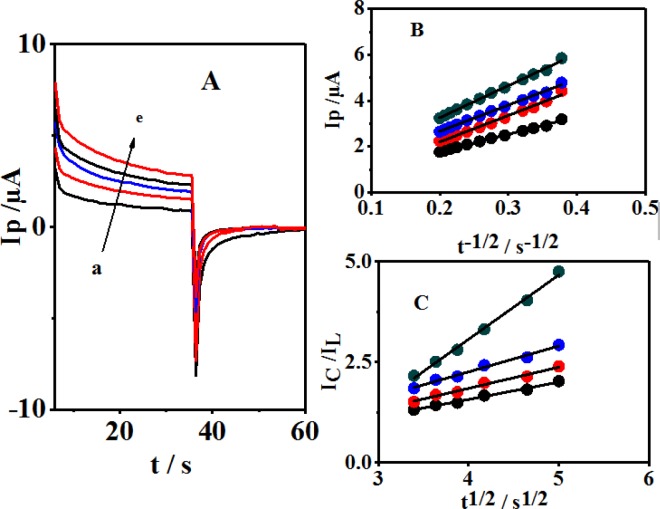
(A) Chronoamperometric responses of a GCE in a buffer solution (pH 10.0) + 0.1 mM 4,4′-biphenol and different amounts of CySH: a: 0.0, b: 40, c: 60, d: 80 and e: 100 μM. (B) Plots of currents versus the square root of time elapsed (t^-1/2^) for the 4,4′-biphenol in (A) b to e. (C) Plots of I_Cat_/I_L_ versus square root of time elapsed (t^1/2^) for the 4,4′-biphenol in (A) b to e.

**Table 1:** Comparison of the results of the proposed method with similar reports for detrminatin of CySH

**Method**	**Electrode**	**Linear range (μmol L** **-1** **)**	**Detection limit (µmol L** **-1** **)**	**Reference**
SWV	Lead ruthenate pyrochlore modified electrode	Up to 560	1.91	(30)
Amperometry	Nafion/PbNP modified carbon ceramic electrode	1-67.2	0.46	(31)
Amperometry	OMC/GCE	18-2500	2	(20)
Amperometry	ZnOx-NP/GCE	0.2-200.0	0.05	(32)
Amperometry	Oxovanadium (IV) complex of salen modified electrode	240-2300	170	(33)
CV	Fullerene-C60-modified carbon electrode	20-300	0.04	(34)
Chronomperometry	MWCNT/Gold nanorods	5–200	0.008	(35)
Amperometry	CPE/PB-AuNP-Pd	0.3–400	0.18	(36)
CV	GCE and mediator 4,4'-biphenol	10-1000	0.99	This work

*CV: Cyclic voltammetry; DPV: Differential pulse voltammetry; ZnOxNP: zinc oxide nanoparticles; OMC: an ordered mesoporous carbon; SWV: Square wave voltammetry.

**Table 2:** Determination of CySH in human serum samples under the optimum conditions (n = 7)

**Concentration Sample**	**Added (μM)**	**Found (μM)**	**Recovery (%)**
Tablet (500 mg)	50	55 ± 0.3	110
Plasma	75	74 ± 0.2	98
Plasma	50	53.5 ± 0.4	107

**Table 3:** Interference of some foreign species on the determination of 40.0 μM CySH under the optimized conditions

**Foreign species**	**Tolerant limits (W** **substance** **/W** **DP** **)**
sucrose, glucose, fructose, L-methionine, L-serine, citric acid, L-histidine, oxalic acid,	850
Ascorbic acid, Uric acid, Acetaminophen	100

## Results and Discussion

*The electrochemical behaviors of the modified electrode*

Figure 1A displays the cyclic voltammograms of 0.1 mM of 4,4′-biphenolat different pH values. As can be seen, 4,4′-biphenolat cyclic voltammogram (B) depicts an anodic (A_1_) and its corresponding cathodic peak (C1) in the positive- and negative-going scans at each pH value, respectively. 

This can be attributed to 4,4′-biphenolat transformation into 4, 4′-diphenoquinone (Q) and vice versa under a quasi-reversible electron-transfer condition (27-28).

Upon increasing the pH, the activation overpotential was seen to reduce and a shift towards negative oxidation peak potentials was resulted due to the participation of protons in the B-to-Q oxidation reaction as follows:

  B Q + ne^-^ + mH^+^                    (1)

where m represents the number of protons taking part in the reaction. The half-wave potential is calculated as follows:

E_1/2 _= Eº_1/2_ – (2.303 mRT/2F) pH                     (2) 

where Eº_1/2 _stands for the half-wave potential at pH 0.0 and m, R, T, F are associated with their typical values. The cyclic voltammogram also demonstrated the average of anodic and cathodic peak potentials as calculated below (29):

E_1/2 _= (Epa + E_pc_)/2                     (3)

This plotting E_1/2 _*vs.* pH can be illustrated through the following Equation 4: 

E_1/2 _= -0.057 pH + 0.705 R² = 0.993                     (4)

Being close to the theoretical value of 59 mV, a slope of 57 mV pH^-1^ within the pH range of 2.0-11.0 suggested the 2-electron and 2-proton processes of the electrode surface reaction as exhibited by Figure 1B, Scheme 1, and Equation 4.

As shown in Figure 2A, the different scan rates of B cyclic voltammograms occur in the presence of CySH in a buffer solution at pH 10.0. According to Figure 2B, a linear relationship exists between the plot of IpA *vs.* v^1/2^ in the range of 5-200 mVsec^-1^. 

Moreover, by increasing the potential scan rate, the peak current ratio (I_pC1_/I_pA1_) is enhanced, which is due to the insufficient reaction time for CySH and B (Figure 2C) (27). Scheme 1 portrays how the process of electron transfer leads to a catalytic reaction.

The current research aimed at examining the cyclic voltammograms of 2 mM of CySH on GCE at a scan rate of 10 mVs^-1^ and a pH value of 10 (Figure 3, Curve a). Figure 3 (Curve c) demonstrates the cyclic voltammogram of 0.1 mM of B at the presence of 2 mM of CySH. After comparing the cyclic voltammograms of B in the presence and absence of CySH (Curve b), a significantly greater anodic peak current (I_pA1_) was clearly observed in the latter case.

The electrochemical behaviors of 0.1 mM of B were investigated in the presence of 0.5 mM of CySH at the scan rate of 10 mVsec^-1^ at different pH values associated with various buffers (2-11) through LSV (Figure 4A). The gradual growth of the oxidation peak current (Ip_A1_) induced by pH enhancement up to a value of 10.0 and its subsequent reduction are displayed in Figure 4B. It was expected that the raised pH level of the solution provoke the anodic peak potential (E_pA1_) shift towards negative potentials (Figure 4C) (29).

*Analytical measurements*

Figure 5A shows the cyclic voltammograms of B at various concentrations of CySH oxidation on GCE for the investigation of the electrocatalytic activity of B. Figure 5B shows the relevant calibration curve. As mentioned above, the anodic peak current (I_pA1_) was increased by CySH concentration enhancement, which further showed a linear calibration range of 10-1000 μM with R² = 0.993. This indicated the complete fitness of the regression line to the experimental data. Therefore, the regression equation (I/µА = 0.005[CySH] + 0.484) could be used for determining the unknown samples. Detrmination of CySH Limit of Detection (LOD) was based on LOD definition of 3 Sb/m, in which S_b _is the blank Standard Deviation (SD) (n = 7) and m is the calibration graph slope. Consequently, CYSH LOD was found to be 0.99 μM. Furthermore, its reproducibility was measured to be 100 μM based on the cyclic voltammograms. The Relative Standard Deviation (RSD) was calculated to be 2.5% for 7 replicates.

The voltamvmetric determination of CySH is done by comparing this approach with the previously reported methods as shown in Table 1. Our analytical parameters of CySH determination clearly demonstrated comparable or better results than those of the previous reports.

*Chronoamperometric studies*

In addition to the above methods, the chronoamperometry method was utilized for studying the electrochemical behaviors of CySH in 4,4′-biphenol (B) solution at the GCE. Figure 6A shows double-step chronoamperograms both in the absence (a) and presence of CySH, representing the mediator (4,4′-biphenol (B) solution) at the GCE. The potential steps applied in this study included the values of 300 and -10 mV *vs.* Saturated Calomel Electrode (SCE). These selected potentials were more positive and negative than the anodic and cathodic peak potentials when completing the oxidation and reduction processes at the electrode surface, respectively, while both the anodic and cathodic reactions could be controlled in the diffusion process. The presence of CySH leads to a reduced cathodic current corresponding to the decrease in 4,4′-biphenol (B). Furthermore, a linear dependency was observed when plotting the net current *vs.* the negative square root of time.

Therefore, the main process of the diffusion control was established. The diffusion coefficient value (D) was obtained by using the slope of the following line based on Cottrell′s equation (Figure 6B) (29):

I = nFAD^1/2^Cπ^−1/2^t^−1/2^                      (5)

where D and C denote the diffusion coefficient (cm^2^ sec^-1^) and bulk concentration (mol cm^−3^), respectively. The diffusion coefficient of CySH was calculated to be 8.79 × 10^-6^ cm^2^ sec^–1^. In addition, the catalytic rate constant (k) was measured for the chemical reaction of CySH with 4,4′-biphenol through the chronoamperometric analysis (27-28):

I_C_/I_L_ = π^1/2^ γ^1/2^ = π^1/2 ^(kC_0_t)^ 1/2^                     (6)

where I_C_ and I_L_ stand for 4,4′-biphenol (B) currents in the presence and absence of CySH, respectively; k represents the catalytic rate constant (mol^–1^ L sec^–1^); t shows the elapsed time (s); and C_0_ demonstrates bulk cysteine concentration (mol L^–1^). A linear dependency was shown by plotting the current ratio (I_C_/I_L_) in relation to the square root of time. k_cat_ value was measured to be 6.17 × 10^3^ M^-1^ sec^-1^ based on the slope of this line (Figure 6C).

Measurements of CySH concentrations in human plasma and commercial tablets were done to determine the applicability of CySH as an electrochemical sensor. CySH can be simply and rapidly assessed via the standard addition method.

The practical usefulness of the prepared CySH sensor was evaluated by analyzing it in various human plasma samples and commercial tablets. A specified amount of CySH was used in the spiking drugs to measure its recovery in the samples and tablets. As displayed in Table 2, the analysis results of CySH contents in the real samples revealed that 4,4′-biphenol (B) could serve as a relatively good redox mediator for the voltammetric determination of CySH in the different matrices of the real samples, indicating the reasonable rates of recovery and reproducibility. 

*Interference studies*

The major challenge in interference studies is that the presence of ascorbic acid (AA) and uric acid (UA) makes it difficult to determine CySH. The selectivity of the prepared sensor was evaluated in the presence of 4,4′-biphenol considering its response to AA, UA, and the materials listed in Table 3, in which the tolerance limit was defoned as a range of relative errors in determining the maximum concentration of troublesome materials, which fall around 5 percent. The results indicated that 40 μM CySH was just the response detected to the prepared sensor, without any trace of the other species.

## Conclusion

In this study, 4,4′-biphenol (B) as a redox mediator was utilized for CySH homogeneous electrocatalysis in an aqueous medium at the GCE surface. As exhibited by Scheme 1, different conditions of 4,4′-biphenol (B) mechanism were assessed for anodic oxidation in the presence of CySH. Cyclic Voltammetry could serve as a proper method in the determination of 4,4′-biphenol electrochemical properties. In the current research, a straightforward method was introduced for describing the second order of the homogeneous catalytic interaction of EC′ between 4,4′-biphenol (B) and CySH as well as identifying 4,4′-biphenol electrochemical properties and determining its effects on CySH oxidation. To achieve the research objectives, CV, LSV, and chronoamperometry techniques were employed. Our findings were indicative of the mediator success in sensitively and selectively determining CySH voltammetry in such real samples as the blood serum and pharmaceutical tablets at low concentrations of the relevant drugs.

## References

[1] Miseta A, Csutora P (2000). Relationship between the occurrence of cysteine in proteins and the complexity of organisms. Mol. Biol. Evol.

[2] Xu J, Wang Y, Xian Y, Jin L, Tanaka K (2003). Preparation of multiwall carbon nanotubes film modified electrode and its application to simultaneous determination of oxidizable amino acids in ion chromatography. Talanta.

[3] Yardim-Akaydin S, Özkan Y, Özkan E, Torun M, Şimşek B (2003). The role of plasma thiol compounds and antioxidant vitamins in patients with cardiovascular diseases. Clin. Chim. Acta.

[4] Bydalek TJ, Poldoski JE (1968). Spectrophotometric determination of cysteine. Anal. Chem..

[5] Bamdad F, Khorram F, Samet M, Bamdad K, Sangi MR, Allahbakhshi F (2016). Spectrophotometric determination of L-cysteine by using polyvinylpyrrolidone-stabilized silver nanoparticles in the presence of barium ions. Spectrochim. Acta A Mol. Biomol. Spectrosc..

[6] Yahong C, Ruxiu C (2003). Highly sensitive spectrofluorimetric determination of l-cysteine based on inhibition of hemoglobin. Spectrochim. Acta A Mol. Biomol. Spectrosc..

[7] Anand T, Sivaraman G, Chellappa D (2014). Hg2+ mediated quinazoline ensemble for highly selective recognition of Cysteine. Spectrochim. Acta A Mol. Biomol. Spectrosc..

[8] Ballin NZ (2006). Estimation of whey protein in casein coprecipitate and milk powder by high-performance liquid chromatography quantification of cysteine. J. Agric. Food Chem..

[9] Chaichi MJ, Ehsani M, Khajvand T, Golchoubian H, Rezaee E (2014). Determination of cysteine and glutathione based on the inhibition of the dinuclear Cu(II)-catalyzed luminol–H2O2 chemiluminescence reaction. Spectrochim. Acta AMol. Biomol. Spectrosc.

[10] Cao ZY, Mou RX, Zhou R, Zhu ZW, Sun LH, Chen MX (2015). A novel method for the simultaneous analysis of seven biothiols in rice (Oryza sativa L) using hydrophilic interaction chromatography coupled with electrospray tandem mass spectrometry. J. Chromatogr. B.

[11] Stein SE (1999). An integrated method for spectrum extraction and compound identification from gas chromatography/mass spectrometry data. J. Am. Soc. Mass Spectrom.

[12] Beitollahi H, Tajik S, Karimi Maleh H, Hosseinzadeh R (2013). Application of a 1-benzyl-4-ferrocenyl-1H-[1, 2, 3]-triazole/carbon nanotube modified glassy carbon electrode for voltammetric determination of hydrazine in water samples. App. Organomet. Chem.

[13] Yang S, Li G, Wang Y, Wang G, Qu L (2016). Amperometric L-cysteine sensor based on a carbon paste electrode modified with Y2O3 nanoparticles supported on nitrogen-doped reduced graphene oxide. Microchim. Acta.

[14] Beitollahi H, Raoof JB, Karimi-Maleh H, Hosseinzadeh R (2012). Electrochemical behavior of isoproterenol in the presence of uric acid and folic acid at a carbon paste electrode modified with 2,7-bis(ferrocenyl ethyl)fluoren-9-one and carbon nanotubes. J. Solid State Chem..

[15] Moghaddam HM, Beitollahi H, Tajik S, Soltani H (2015). Fabrication of a nanostructure based electrochemical sensor for voltammetric determination of epinephrine, uric acid and folic acid. Electroanalysis.

[16] Pourghobadi Z, Neamatollahi D (2017). Voltammetric determination of dopamine using modified glassy carbon electrode by electrografting of catechol. ‎J. Serb. Chem. Soc..

[17] Hosseini H, Ahmar H, Dehghani A, Bagheri A, Tadjarodi A, Fakhari AR (2013). A novel electrochemical sensor based on metal-organic framework for electro-catalytic oxidation of L-cysteine. Biosens. Bioelectron.

[18] Beitollahi H, Ghofrani Ivari S, Torkzadeh-Mahani M (2018). Application of antibody–nanogold–ionic liquid–carbon paste electrode for sensitive electrochemical immunoassay of thyroid-stimulating hormone. Biosens. Bioelectron.

[19] Beitollahi H, Tajik S, Jahani S (2016). Electrocatalytic determination of hydrazine and phenol using a carbon paste electrode modified with ionic liquids and magnetic core-shell Fe3O4@SiO2/MWCNT nanocomposite. Electroanalysis.

[20] Zhou M, Ding J, Guo LP, Shang QK (2007). Electrochemical behavior of L-cysteine and its detection at ordered mesoporous carbon-modified glassy carbon electrode. Anal. Chem.

[21] Beitollahi H, Karimi-Maleh H, Khabazzadeh H (2008). Nanomolar and selective determination of epinephrine in the presence of norepinephrine using carbon paste electrode modified with carbon nanotubes and Novel 2-(4-Oxo-3-phenyl-3,4-dihydroquinazolinyl)-N′-phenyl-hydrazinecarbothioamide. Anal. Chem.

[22] Beitollahi H, Nekooei S (2016). Application of a modified CuO nanoparticles carbon paste electrode for simultaneous determination of isoperenaline, acetaminophen and N-acetyl-L-cysteine. Electroanalysis.

[23] Beitollahi H, Sheikhshoaie I (2012). Electrochemical behavior of carbon Nanotube/Mn(III)salen doped carbon paste electrode and its application for sensitive determination of N-acetylcysteine in the presence of folic acid. Int. J. Electrochem. Sci..

[24] Beitollahi H, Garkani Nejad F (2016). Graphene oxide/ZnO Nano composite for sensitive and selective electrochemical sensing of levodopa and tyrosine using modified graphite screen printed electrode. Electroanalysis.

[25] Ge S, Yan M, Lu J, Zhang M, Yu F, Yu J, Song X, Yu S (2012). Electrochemical biosensor based on graphene oxide–Au nanoclusters composites for l-cysteine analysis. Biosens. Bioelectron..

[26] Bahramipur H, Jalali F (2011). Voltammetric determination of captopril using chlorpromazine as a homogeneous mediator. Int. J. Electrochem. Sci.

[27] Shayani-Jam H, Nematollahi D (2011). Electrochemically mediated oxidation of glutathione and N-acetylcysteine with 4,4′-biphenol. Electrochim. Acta.

[28] Niazi A, Pourghobadi Z, Nematollahi D, Beiginejad H (2014). Electrochemical oxidation and voltammetric determination of captopril using 4,4′-biphenol as a homogeneous mediator. J. Electrochem. Soc..

[29] Bard AJ, Faulkner LR (2001). Electrochemical Methods. 2nd ed. Wiley, New York.

[30] Zen JM, Kumar AS, Chen JC (2001). Electrocatalytic oxidation and sensitive detection of cysteine on a lead ruthenate pyrochlore modified electrode. Anal. Chem..

[31] Razmi H, Heidari H (2009). Nafion/lead nitroprusside nanoparticles modified carbon ceramic electrode as a novel amperometric sensor for l-cysteine. Anal. Biochem.

[32] Hallaj R, Salimi A, Akhtari K, Soltanian, Mamkhezri H (2009). Electrodeposition of guanine oxidation product onto zinc oxide nanoparticles: Application to nanomolar detection of l-cysteine. Sensor. Actuat. B Chem.

[33] Teixeira MFS, Dockal ER, Cavalheiro ETG (2005). Sensor for cysteine based on oxovanadium (IV) complex of Salen modified carbon paste electrode. Sensor. Actuat. B Chem.

[34] Tan WT, Bond AM, Ngooi SW, Lim EB, Goh JK (2003). Electrochemical oxidation of l-cysteine mediated by a fullerene-C60-modified carbon electrode. Anal Chim Acta.

[35] dos Santos Silva FA, da Silva MGA, Lima PR, Meneghetti MR, Kubota LT, Fonseca Goulart MO (2013). A very low potential electrochemical detection of l-cysteine based on a glassy carbon electrode modified with multi-walled carbon nanotubes/gold nanorods. Biosens. Bioelectron..

[36] Pandey PC, Pandey AK, Chauhan DS (2012). Nanocomposite of Prussian blue based sensor for l-cysteine: Synergetic effect of nanostructured gold and palladium on electrocatalysis. Electrochim. Acta.

